# Brain somatic mutations in Alzheimer’s disease: linking genetic mosaicism to neurodegeneration

**DOI:** 10.1186/s13024-025-00895-0

**Published:** 2025-10-09

**Authors:** Zuguang Li, Juan Zhang, Zhiqiang Liu, Lu Yu, Chunqing Yang, Luoman Zhang, Zhigao Xiang, Feng Hu, Nadezda Brazh, Kai Shu, Ling-Qiang Zhu, Dan Liu

**Affiliations:** 1 Department of Pathophysiology, School of Basic Medicine, Tongji Medical College, Huazhong University of Science and Technology, No. 13, Hangkong Road, Wuhan, 430030 China; 2 Department of Endocrinology and Metabolism, Union Hospital, Tongji Medical College, Huazhong University of Science and Technology, Wuhan, 430000 China; 3 Department of Pathology, The First Affiliated Hospital of Zhengzhou University, Zhengzhou, 450002 China; 4 Department of Neurosurgery, Tongji Hospital, Tongji Medical College, Huazhong University of Science and Technology, Wuhan, 430030 China; 5 Faculty of Biology, Lomonosov Moscow State University, Moscow, 119234 Russian Federation; 6 Department of Medical Genetics, School of Basic Medicine, Tongji Medical College, Huazhong University of Science and Technology, Wuhan, 430030 China

**Keywords:** Somatic mutations, Alzheimer’s disease, Environmental exposures

## Abstract

Somatic mutations are DNA sequence changes that occur in non-reproductive cells during an organism’s life and are not inherited by offspring. Growing evidence implicates somatic mutations in Alzheimer’s disease (AD), linking them to both disease onset and progression. Recent advancements in single-cell sequencing and genome-wide analyses have revealed higher mutation burdens in neurons, particularly in AD-related genes such as *Presenilin 1 (PSEN1)*,* Presenilin 2 (PSEN2)* and *amyloid precursor protein (APP)*. These mutations, which include single nucleotide variants (SNVs), small insertions and deletions (Indels), structural variations (SVs) and mitochondrial DNA (mtDNA) mutations may disrupt neuronal function and synaptic connectivity. However, some somatic mutations may also serve a neuroprotective role. The underlying mechanisms remain incompletely understood. This review explores the emerging role of somatic mutations in AD, highlighting their links to disease progression. It also underscores the potential for future research to uncover new therapeutic targets by integrating advanced sequencing technologies and gene-editing approaches, which may enable more precise interventions to correct somatic mutations and slow disease progression.

## Introduction

Epidemiological studies on AD consistently demonstrate its high prevalence and wide spectrum of affected individuals, establishing it as the leading cause of dementia worldwide [1]. Multiple studies have unequivocally identified advanced age as a major risk factor for AD development [2]. Experts currently recognize three pathological hallmarks of AD: amyloid-beta (Aβ) protein deposition [3, 4], hyperphosphorylation of Tau protein [5, 6], and chronic neuroinflammation [7]. These pathological alterations ultimately cause neuronal dysfunction and progressive neurodegeneration [8–10]. A number of groups have identified a cluster of AD-associated genes, including *APP* [11], *PSEN1* [12], and *PSEN2* [13], which play pivotal roles in AD pathogenesis. Specifically, highly penetrant mutations in these genes cause monogenic forms of AD collectively referred to as Familial AD (FAD). FAD typically shows early onset and autosomal dominant inheritance patterns [14]. These genetic discoveries provide crucial insights into the molecular mechanisms underlying AD pathogenesis. In addition to these highly penetrant mutations, geneticists have identified numerous susceptibility genes, whose polymorphisms may influence an individual’s risk of developing AD [15, 16].

The majority of AD cases fall under the category of sporadic AD (SAD), which shows later symptom onset and the absence of familial inheritance patterns [17]. The inability of inherited. As a result, researchers considered the role of somatic mutations in aging and related diseases to be negligible mutations alone to fully account for AD pathogenesis underscores the significant contribution of environmental factors to disease development [18]. Growing evidence shows that diverse environmental exposures can induce somatic mutations via multiple molecular pathways [19]. Researchers first proposed the hypothesis in 2004 that somatic mutations contribute to sporadic neurodegenerative diseases, laying the foundation for subsequent research [20].

Somatic mutations, distinct from both de novo mutations [21] and germline mutations [22], refer to DNA sequence alterations that occur in non-reproductive cells during an organism’s growth and aging. While these mutations are not inherited by offspring, they can significantly influence cellular physiology and function. Recent advances in sequencing technologies—particularly in throughput and accuracy—reveal that somatic mutations are prevalent not only in pathological conditions but also in normal cell populations [23]. These mutations are broadly classified into four categories: SNVs, Indels, and SVs, including insertions, deletions, duplications, recombinations, translocations, and chromosomal losses and gains [24], , and mtDNA mutations.

In the field of oncology, it has identified numerous clinically significant somatic mutations, particularly in driver genes such as *EGFR*, which play pivotal roles in tumor initiation and progression [25]. Unlike most somatic cells, neurons in the human CNS possess limited regenerative capacity, with neurogenesis primarily confined to the sub-ventricular zone (SVZ) and sub-granular zone (SGZ) [26]. This unique characteristic means that somatic mutations in neurons cannot arise via extensive postnatally occurring cell divisions. Furthermore, because neurons are post-mitotic, they cannot propagate somatic mutations through DNA replication, resulting in exceptionally low variant allele frequency (VAF). This low VAF makes somatic mutations in neurons highly susceptible to background noise, posing significant challenges for detection.

It has increasingly recognized that the importance of somatic mutations in CNS pathology, with numerous studies establishing compelling associations between these mutations and various neurodegenerative disorders. For instance, somatic *BRAF* ^*V600E*^ mutations in erythro-myeloid progenitor lineages have been implicated in neurodegenerative processes [27]. It has widely believed that the somatic expansion of the mutated CAG repeat sequence in brain cells marks the first step in the molecular pathogenesis of Huntington’s disease [28]. Recent studies have used integrated profiling of CAG repeat lengths and transcriptomes in caudate nucleus neurons to delineate the relationship between CAG repeat dynamics and disease progression [29]. Despite these advances, it has poorly understood the relationship between somatic mutations and AD, one of the most prevalent neurodegenerative disorders.

This review highlights recent advances in our understanding of somatic mutations in brain cells and emphasizes their multifaceted roles in CNS degeneration, especially in AD. We explore the diverse factors and underlying mechanisms that drive the accumulation of somatic mutations in neural cells. By elucidating the physiological and pathological implications of these genetic alterations, we aim to provide deeper insight into AD pathogenesis through the lens of somatic mutagenesis and to propose new avenues for therapeutic intervention and diagnostic development.

## Types of somatic mutations

### SNVs

Scientists define a SNV as the substitution of a single nucleotide at a specific site in the genome [30]. SNVs occur ubiquitously across various cell types, with neural cells exhibiting a substantial number of somatic mutations [31–34]. Several studies show that hundreds of SNVs exist in each neuron [35]. After excluding clonal mutations arising during neurodevelopment, it has demonstrated that human neurons accumulated approximately 16.5 SNVs per year on their autosomes [36]. Moreover, the SNVs burden in neurons across multiple brain regions strongly correlates with age. Categorizing mutation sites and their flanking base contexts into distinct mutational signatures facilitates identifying potential triggers for point mutations [37–39]. A concise summary of brain-relevant SNV signatures and their cell-type distributions is provided in Table 1.

Neurons in the prefrontal cortex (PFC) and dentate gyrus (DG) display abundant C > T mutations that primarily form Signature B [40]. This mutation occurs widely across biological contexts [41–43]. It has suggested that mutation might result from either the deamination of methylated cytosine in non-replicating neurons or cell division processes [44–46]. Alternatively, multiple displacement amplification (MDA) could produce this mutation as an artifact [47]. Notably, Signature B somatic mutations peak in infancy and are particularly concentrated in the DG, where neurogenesis occurs, indicating a potential link to DNA replication [48]. Distinguishing true mutations from amplification artifacts in single-cell sequencing is therefore crucial. Primary template-directed amplification (PTA) has been shown to substantially reduce amplification artifacts, improving the detection of genuine somatic mutations arising during developmental mitosis and spontaneous deamination [36].

A distinctive “clock-like” mutation pattern, designated as Signature A, occurs in neurons. This signature mainly consists of C > T and T > C mutations and resembles single base substitution (SBS) 5 [40, 49]. Although its mechanism remains unclear, Signature A mutations gradually accumulate with age. Importantly, this mutation appears universally in neurons, independent of brain region or disease state [40], and is also found widely in other cell types [19].

The most critical mutation involves the C > A substitution, known as Signature C. Due to its high cosine similarity with SBS18, which associates with oxidative damage [50], it has speculated that this substitution related to guanine nucleotide oxidation [40]. When 8-oxoguanine (8-oxoG), the oxidation product of guanine, is not promptly repaired, it can mispair with adenine, causing C > A mutations [51].

In oligodendrocytes (OLs), besides SBS5—which resembles that seen in neurons—mutations such as SBS1 and SBS32, less common in neurons, also occur [33, 52]. Similarly, the somatic mutation profile of microglia differs from that of neurons, with SNVs in cancer-related genes significantly enriched in microglia [34]. This highlights the substantial differences in somatic mutations across different cell types in the CNS, underscoring the necessity of accurately identifying somatic mutation profiles in various cell types, or even in individual cells.

### Indels

The COSMIC database defines Indels as mutation types affecting fewer than 50 bp (Table 1) [53]. In both OLs and neurons, the burden of Indels linearly with age [33]. Studies reported that neurons could accumulate approximately 3 Indels per year (with deletions being more frequent than insertions), while OLs exhibit a slightly lower accumulation rate than neurons [36]. Elderly individuals typically harbor 200–300 Indels in their neurons, with some pathological neurons even displaying thousands of Indels [54].

Neuronal Indels can be classified into two major categories: ID-A and ID-B. ID-A, predominantly characterized by 2-bp deletions, is strongly associated with various neurodegenerative diseases, likely reflecting TOP1-mediated defective ribonucleotide repair [54]. This signature resembles ID4 in the COSMIC database—a mutation pattern frequently observed in cancers and linked to TOP1-dependent rNMP repair deficiency [55]—and has been detected exclusively in neurons [54, 56, 57]. By contrast, ID-B appears unrelated to disease and has not been extensively studied. In non-repetitive sequences, ID22 has also been reported to contribute to these 2-bp deletions [56]. In OLs, the most prevalent signature is ID9, characterized by 1-bp deletions [33], ID9 is commonly observed in gliomas and other brain tumors [58, 59]. Moreover, “clock-like” deletion signatures, ID5 and ID8, are present in both neuronal and glial populations [33].

### SVs

It has defined SVs as DNA alterations involving sequences longer than 50 base pairs, including insertions, deletions, duplications, recombinations, translocations, and chromosomal losses and gains, which are summarized in Table 1 [60]. Studies have reported that each neuron undergoes at least three insertion and deletion events annually [36], and that deletions occur more frequently than duplications in neurons [61, 62]. Early studies found that frontal cortical neurons exhibit increased DNA content, averaging approximately 250 Mb per neuron (about 4% of the genome) [63]. Such extensive genomic alterations can’t be attributed to SNVs or Indels, necessitating focused investigation on larger-scale SVs.

The spectrum of SVs varies widely. In addition to nanoscale deletions resulting from non-homologous end joining (NHEJ) repair of double-strand breaks (DSBs) [64], it has identified larger de novo SVs. Among these, megabase-scale SVs have drawn particular attention, as studies have reported highly variable frequencies of neurons carrying such alterations (ranging from 9% to 41%) [65, 66]. Further studies have shown that neurons with megabase-scale de novo SVs and highly aberrant karyotypes populate the neurotypical human brain [62], with notable inter-individual variability [61]. The absence of megabase-scale SVs in fetal brains suggests these mutations likely occur during late developmental stages or in postnatally matured neurons [67]. Non-allelic homologous recombination (NAHR)—a prevalent mechanism involving repetitive elements and DSBs—has emerged as a major contributor to SVs formation [68, 69]. Pascarella et al. demonstrated that NAHR occurs frequently in neurons, averaging approximately 1.4 events per cell, with its recombination spectrum influenced by differentiation status and disease conditions [70].

Researchers classify chromosomal numerical alterations—such as aneuploidy, which involves the gain or loss of entire chromosomes—as a larger category of SVs [71]. Reported frequencies of neuronal aneuploidy vary substantially (ranging up to 40%, with an average of ~ 10%) [72–74]. However, recent single-cell sequencing studies revealed that aneuploidy was rare in frontal cortical neurons from both AD patients and healthy controls [75], likely because such large-scale genomic changes result in cell death. Interestingly, the Y chromosome, which carries relatively limited genetic information, could persist and accumulate in certain male cells [76]. Subsequent investigations showed that mosaic Y chromosome loss predominantly occurs in microglial cells and was exceptionally rare in other brain cell types [77].

It has shown that transposable elements (TEs), which were abundant in eukaryotic genomes, generate substantial structural variations. These elements fall into Class I and Class II. Class I includes long terminal repeat (LTR) and non-LTR retrotransposons, which relocate to new positions upon activation [78]. It is well-established that the retrotransposon *LINE1 (L1)*, through its transposition activity, leads to gene insertions, occurring not only during embryonic and neural progenitor cell development [79, 80], but also in mature, non-dividing neurons when engineered L1 elements are effectively retrotransposed [81]. However, Limited evidence supporting successful transposition of *L1* in mature neurons. Richardson et al. conducted a comprehensive study on the mechanisms, functions, and pathology of *L1* transposition in the brain [82]. Similarly, Peze-Heidsieck et al. described the lifecycle of *L1* retrotransposition and the factors that influence this process [83]. Different research groups have used distinct sequencing methodologies and reported substantially varying frequencies of *L1* transposition events in neurons. For instance, Upton et al. reported an average of approximately 13.7 *L1* transpositions per hippocampal neuron [84]. In contrast, Evrony, Sanchez-Luque, and colleagues found virtually no evidence of *L1* transposition in either hippocampal or cortical neurons [45, 85, 86].

### mtDNA mutations

Mitochondria contain a circular genome of 16,569 base pairs, encoding 13 proteins essential for the respiratory chain [87]. Scientists classify mutations in mtDNA as either heteroplasmic, affecting only a subset of mtDNA copies within a cell, or homoplasmic, impacting all copies [88]. Due to its high exposure to reactive oxygen species (ROS) [89], the lack of histone protection, and absence of mismatch repair and nucleotide excision repair mechanisms, mtDNA exhibits a mutation rate 100–1,000 times higher than that of nuclear DNA (nDNA) [90]. Notably, mtDNA mutations differ significantly from those in nDNA: the transition-to-transversion ratio is markedly higher in mtDNA, and mutations show a distinct strand bias favoring the heavy strand [46, 91]. Recent studies have identified reduced proofreading activity of DNA polymerase γ (DNA pol γ) as a key driver of mtDNA mutations in both mouse and human cells [92]. Key features are summarized in Table 1.

In the brain, particularly within the cortex, aging leads to an increased mtDNA mutation burden and heteroplasmy [93–95]. This accumulation primarily involves G > A and T > C transitions [96], which are likely driven by cytosine deamination, adenosine deamination, and errors introduced by DNA pol γ [97, 98]. These findings support the growing view that the intrinsic error rate of DNA pol γ plays a major role in generating mtDNA mutations [99]. It has reported that elevated oxidative stress couldalter the structure of the exonuclease domain of DNA pol γ, reducing its exonuclease activity and consequently impairing replication fidelity [100]. Studies showed that DNA pol γ expression decreases following exposure to Aβ amyloid plaques [101], which might partly explain the high frequency of G > A and T > C transitions observed in neurodegenerative conditions. Surprisingly, researchers have found that mutations indicative of direct oxidative damage, such as C > A, could not significantly present in either AD patients or healthy individuals [96, 102]. A similar phenomenon has been reported in tumor cells [103]. One possible explanation was that 8-oxoG suppresses DNA replication [104], leading to mitochondrial depletion—a process frequently observed in aging and AD [95, 105]—rather than mutation accumulation. Alternatively, mitochondria might possess highly efficient antioxidant systems that limit oxidative mutagenesis.

**Table 1:** Common somatic mutation types and their landscape in brain cells

Types of somatic mutation	Definition	Somatic mutation landscapes in brain cells
**Single nucleotide variants**	Single nucleotide replacements (transition or transversion) [30]	Most common mutation type; hundreds per neuron [30] ; ~16.5 new per year excluding clonal mutations [36] ; microglia mutations enriched in tumor-driving genes [34]
Signature A	A Mutation pattern mainly C > T and T > C [40]	Accumulates with age; independent of brain regions or pathology [40].
Signature B	Mutation pattern mainly C > T [40]	Related to cell division, cytosine deamination, and sequencing artifacts [44–46]; enriched in dentate gyrus and infant neurons [48]
Signature C	Mutation pattern mainly C > A [40]	Linked to oxidative damage; higher in Alzheimer’s disease neurons [40]
SBS1	Mutation pattern mainly C > T [106]	Accumulates with age; Linked to cell division [107]; common in OLs and microglia, but rare in neurons [33, 34]
SBS32	Mutation pattern mainly C > T [33]	Accumulates with age; common in OLs, but rare in neurons [33]; enriched in LINE-1 elements [108]
SBS16	Mutation pattern mainly T > C [109]	Associated with alcohol consumption [109]; mainly present in neurons; characterized by transcription-associated features [33]
**Small insertions and deletions**	Insertions/deletions < 50 bp [53]	Occur across brain cells; neurons accumulate ~ 3 per year [33].
ID4 (a major contributor to ID-AD) [56]	Dinucleotide deletions [54]	Predominantly in neurons [54, 57]; elevated in neurodegenerative disorders [54].
ID9	Single-nucleotide deletions [33]	Mainly in glial cells [33]; common in gliomas and brain tumors [55, 56].
ID22 (a contributor to ID-AD) [56]	2/3 bp deletions enriched in non-repetitive sequences [56]	Etiology unclear; AD-specific; minimal in control neurons [56]
**Structure variations**	Genetic changes > 50 bp [110]	Present in neurons, microglia, OLs [33, 34, 111]
Copy number variations	DNA fragments 1 kb to several Mb with altered copy number (include insertions and deletions) [61]	Common in neocortical neurons (13.1%); larger and more frequent than in non-neuronal cells [61]
Transposon insertion	Movement of transposable elements by copy/cut-paste [78]	Frequent in embryonic and progenitor cells [79, 80] ; controversial in mature neurons [45, 84–86]
NAHR	DSB repair using non-allelic homologous sequences [68, 69]	Avg 1.4 events/cell [70] ; no difference between neurons and others; linked to differentiation and pathology [70]
Aneuploidy	Whole chromosome gains or losses [71]	Controversial burden [72–74]; Y chromosome loss confirmed mainly in microglia [77]
**Somatic mutations in mtDNA**	Mutations in mitochondrial DNA [90]	Frequent due to lack of protection; 100-1000x mutation rate of nuclear DNA [90]
Oxidative damage point mutations in mtDNA	G: C > T:A transitions caused by ROS-induced 8-oxoG mispairing [96, 102].	Small fraction of mtDNA mutations [96, 102].
Other mtDNA point mutations	G: C > A:T and T: A > C:G transitions [96].	Major component caused by cytosine/adenine deamination and DNA pol γ errors [97, 98].

## Causes and regulatory factors of somatic mutations

### Environmental and lifestyle factors influencing somatic mutations

Exposure to heavy metals such as arsenic, cadmium, and mercury increases the risk of AD. Air pollution, especially fine particulate matter (PM2.5), also represents a major environmental risk factor for AD [112]. Regular physical activity reduces the risk of AD and slows cognitive decline in affected individuals [113, 114]. Sufficient intake of vitamin B12 and folate correlates with lower AD risk [115]. Epidemiological data also reveal a strong association between pesticide exposure—particularly organophosphates—and increased AD risk [116], with a notably higher incidence in men [117].

Although several studies have examined how these factors influence AD progression, the underlying mechanisms remain incompletely defined. For example, lead, cadmium, and organophosphate pesticides intensify neuroinflammation [118–120], whereas vitamin B12 and folate reduce such inflammation [121, 122]. Physical activity improves mitochondrial structure and function, providing neuroprotective effects [123–125]. However, current evidence remains fragmented, and a complete mechanistic understanding is still lacking.

We propose that these environmental and lifestyle factors may promote somatic mutations. For example, nickel chloride has been shown to reduce DNA repair activity, thereby increasing the frequency of *L1* retrotransposition [126]. Metals such as chromium, cadmium, and arsenic induce oxidative stress and DNA repair defects following DNA damage, leading to genomic instability and mutations [127]. Notably, the activity of RNase H2—an enzyme closely associated with Indels formation discussed later—is regulated by divalent metal ions such as calcium and magnesium [128]. Even extremely low concentrations of cesium and lithium salts, as well as cellular senescence, have been shown to suppress RNase H2 activity, potentially leading to an increased Indel burden [129, 130]. Benzo(a)pyrene, a major component of PM2.5, induces DNA damage and activates *L1* retrotransposition [131]. Both air pollution and arsenic exposure have been linked to mosaic loss of chromosome Y (mLOY) in male leukocytes [132, 133]. Vitamin B12 and folate levels are associated with proper DNA replication and repair mechanisms, limiting uracil misincorporation and maintaining genomic stability [134]. Both deficiencies and excesses of these nutrients have been shown to increase mutation rates and heterogeneity in mtDNA [135]. Organochlorine pesticides may induce DNA damage either through oxidative stress or direct interaction with DNA [136, 137]. It is hypothesized that chronic low-dose exposure to these pesticides induces non-lethal mutations that accumulate in terminally differentiated neurons [138]. Furthermore, lifestyle factors and nutrients have been shown to influence *L1* methylation, with methylation regulating *L1* activation [139, 140]. Prolonged physical activity reduces DNA damage and enhances antioxidant capacity [141].

Although the phenomena described above have not been directly observed in the brain, many of the influencing factors can enter systemic circulation through the skin, lungs, and gastrointestinal tract and subsequently cross the blood-brain barrier, potentially disrupting brain cell function [142, 143]. Therefore, we hypothesize that similar processes likely occur in brain cells.

**Fig. 1:**
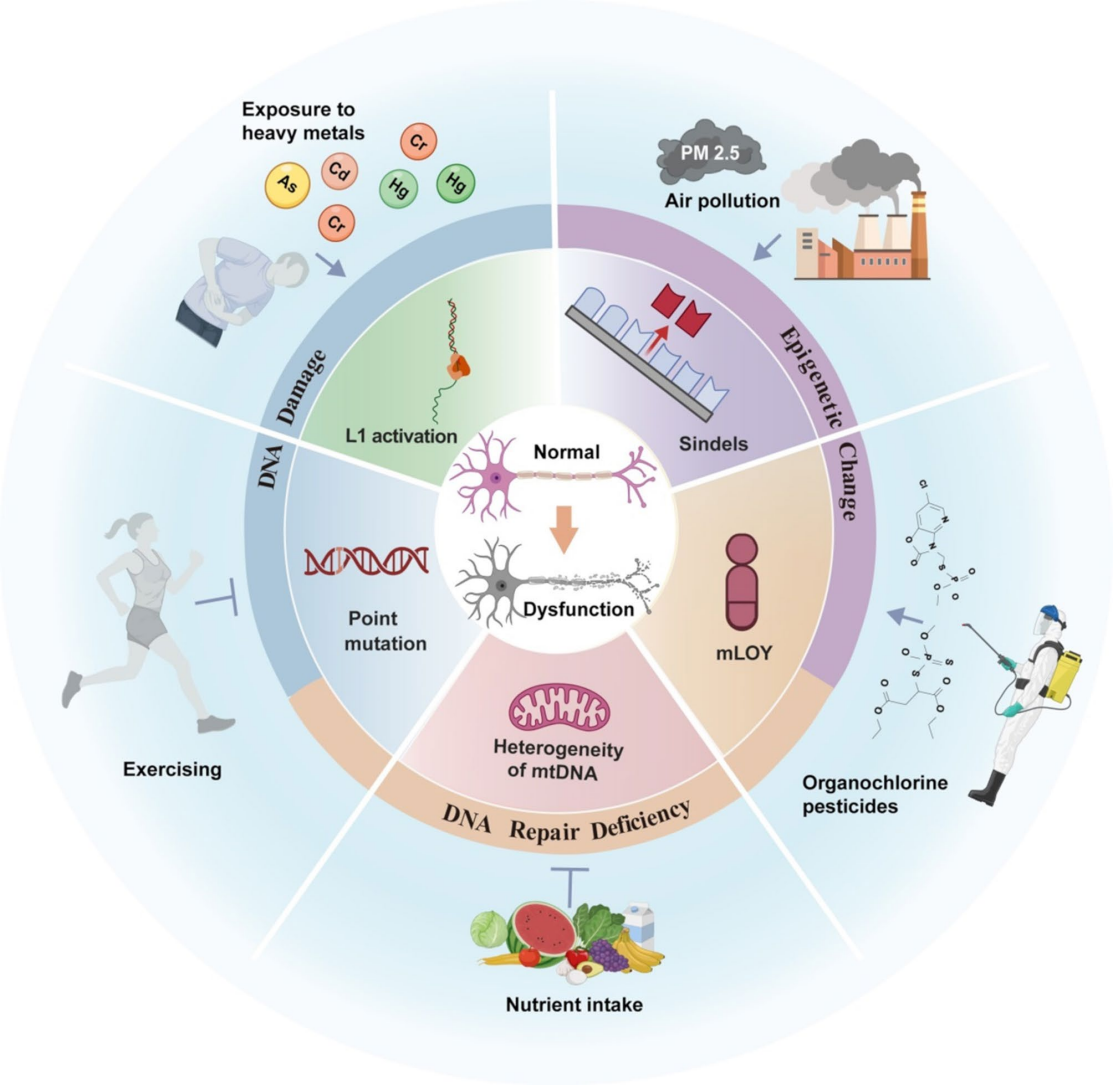
Environmental and lifestyle factors influencing somatic mutations

In summary, environmental factors may contribute to somatic mutations by damaging DNA, impairing DNA repair mechanisms, and altering the epigenetic landscape (Fig.1). Clarifying these processes will help elucidate the link between environmental exposures and AD through the lens of somatic mutagenesis.

Heavy metals, particulate matter in air pollution, and organophosphorus pesticides promote somatic mutations through DNA damage, inhibition of DNA repair, and alteration of epigenetic mechanisms. The body primarily manifests as neuroinflammation, activation of ROS, and genomic mutations, including increased frequencies of mLOY, heightened heterogeneity in mtDNA, elevated L1 retrotransposon activity, and increased frequencies of small-fragment DNA deletions. These mechanisms collectively impair neuronal survival and affect microglial function, thereby accelerating the onset and progression of AD. Conversely, the intake of nutrients such as vitamin B12 and folic acid, along with physical exercise, can alleviate DNA damage, promote DNA repair, and suppress somatic mutations through epigenetic modifications, thereby mitigating the pathological development of AD.

### Molecular mechanisms underlying somatic mutations

#### DNA damage

Due to the robust blood-brain barrier, which prevents the entry of the most genotoxic substances, endogenous ROS emerge as the primary DNA damage factor in the brain [144]. Oxidation not only directly damages DNA, leading to somatic mutations, but also compromises replication fidelity by impairing DNA polymerase γ [100]. The increase in ROS and oxidative DNA damage during the aging process is well-documented [145, 146], and in AD, oxidative stress is already evident prior to the onset of Aβ pathology [147].

Neurons are particularly susceptible to DNA damage and frequently sustain DSBs and other lesions [148]. There is substantial evidence supporting an increased incidence of DSBs in patients with Mild cognitive impairment (MCI) and AD [149–151]. Although DSBs are essential for neural development and gene regulation [148], they can also trigger aberrant repair mechanisms, leading to somatic mutations associated with DNA rearrangements [64, 152]. The loci where gene fusions occur tend to exhibit a higher frequency of DSBs [111], suggesting a potential association between DSBs and the occurrence of gene fusion.

Another common form of DNA damage is ribonucleotide misincorporation (rNMP). This primarily arises because the intracellular concentration of ribonucleoside triphosphates (rNTPs) far exceeds that of deoxyribonucleoside triphosphates (dNTPs), leading to polymerase misselection [153]. Moreover, studies have shown that rNMP incorporation occurred at high frequency during the NHEJ repair process [154]. MtDNA generally tolerates rNMPs relatively well [155]. However, when large amounts of rNMPs are incorporated into the nuclear genome, they can exert various deleterious effects [156, 157], including the formation of Indels [158]. The generation of somatic mutations from rNMP incorporation is also closely linked to topoisomerase I (Top1)-mediated processing.

#### DNA repair

**Fig. 2:**
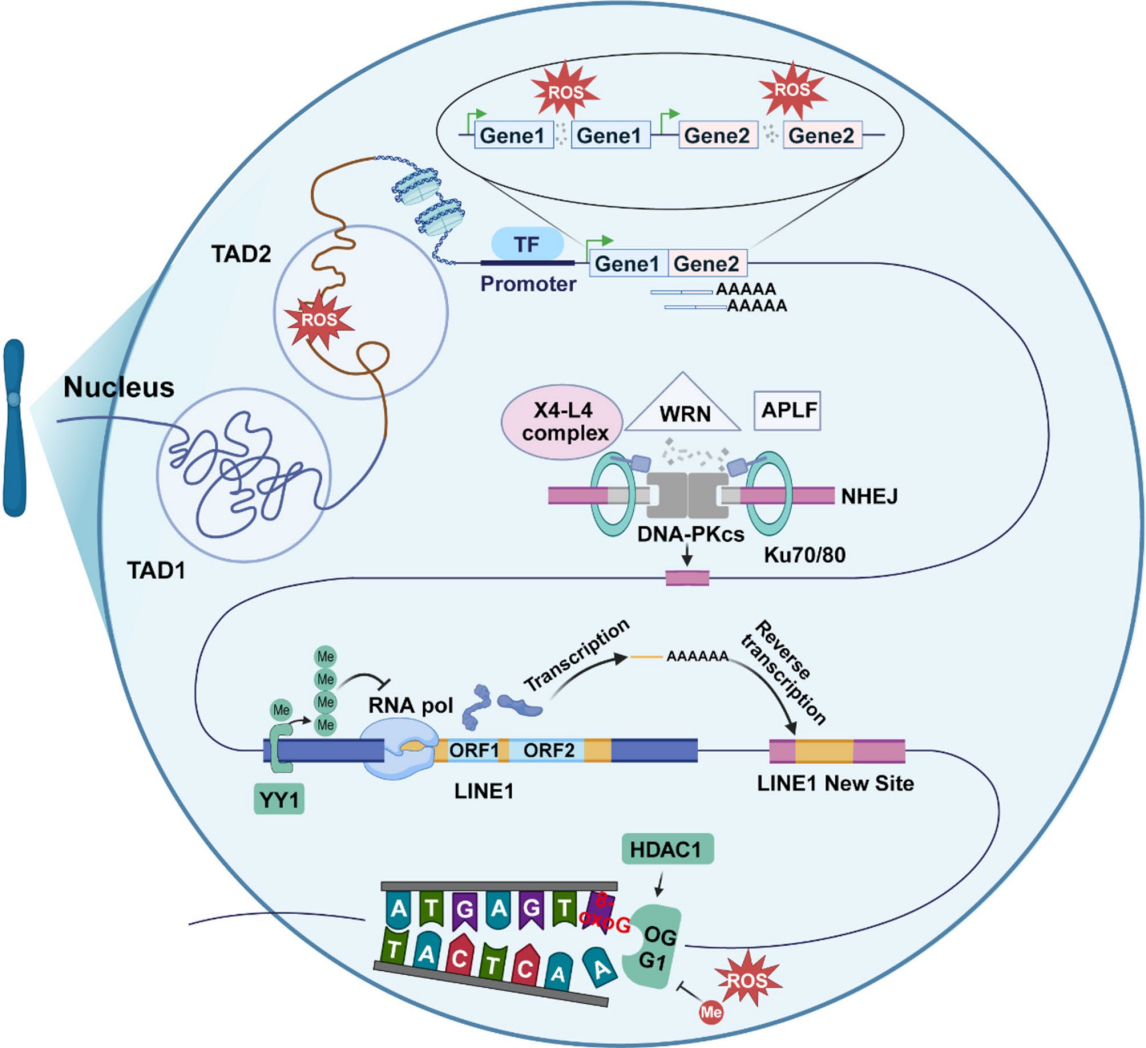
Mechanisms of somatic mutation induced by ROS

Under physiological conditions, a dynamic equilibrium is maintained between DNA damage and repair to preserve genomic stability. Neurons, which lack proliferative capacity, heavily rely on DNA repair to ensure genomic accuracy. Researchers estimate that a single neuron may undergo up to a billion DNA repair events during a person’s lifetime [159]. When the extent of damage exceeds the repair capacity, genetic mutations and apoptosis can occur, phenomena that become increasingly prevalent in senescent cells [160]. Neurons possess a robust DNA repair system to promptly address various DNA damages and prevent mutations. Single-cell sequencing of neurons from individuals with conditions such as xeroderma pigmentosum (XP), Cockayne syndrome (CS), and ataxia-telangiectasia (AT) — all characterized by DNA repair deficiencies — has revealed a significantly higher rate of somatic mutations compared to normal individuals [64]. These repair abnormalities contribute to accelerated aging and AD, in addition to diseases such as skin cancer [161]. Research on DNA repair in AD has primarily focused on the base excision repair (BER) pathway, which addresses base damage from oxidation and alkylation. N-glycosylase/DNA lyase (OGG1) is a key glycosylase involved in this pathway (Fig.2) [162]. Clinical data examining blood and brain tissues suggest that variations in BER-related genes may be associated with an elevated risk of SAD in non-APOEε4 carriers [163].

The BER pathway involves the recognition and excision of specific base modifications by glycosylases, such as OGG1. However, the limited number of glycosylases in the human genome makes the BER pathway insufficient to repair the full spectrum of DNA damage [164]. Another crucial repair mechanism in the human body is nucleotide excision repair (NER), a versatile pathway capable of repairing a variety of DNA lesions [165]. The capacity of NER increases during cellular differentiation, underscoring its importance in post-mitotic cells [166]. Although research is limited, recent studies indicate that NER may be involved in the pathology of chronic neurodegenerative diseases such as AD [167].

Interestingly, it has found that imperfect DNA repair mechanisms in neurons could directly cause somatic mutations. For example, in the NHEJ pathway, which repairs DSBs, Ku70/80 initially binds to the DNA ends. DNAPKcs then trims the ends until DNA single strands with homologous sequences are exposed, after which repair is facilitated by molecules such as the X4-L4 complex, WRN, and APLF [168]. This repair process can result in structural variations, including gene fusions (Fig.2) [111].

In addition to the mutation burden, the loss of ataxia-telangiectasia mutated (ATM) protein, a serine/threonine kinase that responds to DNA damage and coordinates cellular processes such as cell cycle arrest, DNA repair, and apoptosis, has been associated with longer *L1* insertions [169, 170]. Moreover, mtDNA repair, like genomic DNA repair, is impaired in AD [171].

In eukaryotic cells, RNase H2 rapidly recognizes rNMPs through its interaction with proliferating cell nuclear antigen (PCNA) [172]. These rNMPs are removed via the highly conserved RNase H2–dependent ribonucleotide excision repair (RER) pathway [173, 174]. When this repair pathway is impaired (e.g., due to RNase H2 deficiency [175]), Top1 can recognize unrepaired rNMPs and introduce nicks into the DNA [176]. These nicks may then be mis-joined, leading to DNA backbone rearrangements [177, 178] and resulting in small deletions of 2–5 base pairs [179, 180]. Reijns et al. demonstrated that such deletions constitute ID4, a somatic mutation signature specifically enriched in neurons and AD, as previously mentioned [55]. Interestingly, this Top1-mediated process may also lead to DSBs—another form of DNA damage discussed earlier [181].

#### Epigenetics

Epigenetics plays a key role in regulating the occurrence of somatic mutations through various mechanisms. Transposons are tightly regulated, and only those that escape genomic defense mechanisms can remain active [182]. In a normal organism, several epigenetically regulated mechanisms control transposon activity. For example, MeCP2 modulates neuronal *L1* retrotransposition by regulating the methylation of the *L1* 5’-untranslated region (UTR) [183]. Recently, Yin Yang 1 (YY1) was discovered in hippocampal neurons, where it binded to the 5’ end of *L1*, potentially initiating high methylation of its promoter by recruiting DNA methyltransferases [184], thereby suppressing its transpositional activity (Fig.2). Transposition efficiency increases with 5′ end truncation and mutations at regulatory binding sites. Nanopore sequencing can detect TE methylation and mobilization, providing a tool for studying these processes in greater detail [185].

Multiple factors in AD influence transposition through epigenetic regulation. It has been reported that pathogenic Tau in fruit flies could activate retrotransposon elements [186]. The underlying mechanism may involve Tau promoting chromatin relaxation [187], or Tau-induced reduction in PIWI/piRNA, which leads to the upregulation of retrotransposon element mRNA [188]. A recent study also indicates the upregulation of histone demethylase KDM4B in the brains of AD patients [189]. This enzyme has been shown to enhance *L1* expression, increase its copy number, and boost transposition efficiency, while its loss results in decreased *L1* expression [190]. Conversely, SIRT6, a histone acetyltransferase, is downregulated in AD [191]. It inhibits *L1* activity by ribosylating KAP1, a corepressor of *L1* [184, 192]. However, this evidence remains indirect, as the activation of TEs and their subsequent transposition involve complex processes of assembly, transport, and integration [193].

In conclusion, increased DNA damage, DNA repair activity, and epigenetic alterations collectively shape somatic mutation patterns. Recent sequencing has identified a fusion mutation in *TP53*, a key DNA repair regulator [111]. This finding suggests that other genes involved in DNA damage response, repair, and epigenetic control also undergo somatic mutations. These interconnected processes and the resulting somatic mutations constitute a vicious cycle that perpetuates genomic instability. The interplay among DNA damage, repair, and epigenetics creates a complex regulatory network underlying somatic mutations in neurodegenerative diseases. Elevated ROS, a known contributor to aging and AD [145, 147], impairs DNA repair not only by directly damaging DNA but also through epigenetic silencing of repair genes. For instance, DNA methylation suppresses *OGG1* expression and further reduces repair efficiency [194]. These findings underscore the intricate interplay between DNA damage, repair mechanisms, and epigenetic regulation in the context of somatic mutations. Understanding this dynamic network provides critical insights into the molecular pathology of neurodegenerative diseases and highlights potential therapeutic targets for mitigating disease progression.

ROS are major contributors to DNA damage, leading to DSBs and base oxidation. When DSBs occur in two genes that are in close spatial proximity, erroneous DNA repair may lead to gene fusion between the two genes. DSB-induced NHEJ repair causes deletion mutations, while the oxidation of guanine to 8-oxoG leads to mispairing with adenine and the introduction of point mutations. ROS also inhibit the activity of OGG1, impairing the repair of 8-oxoG. Additionally, DNA methylation levels affect the transcription of *L1* elements, thereby controlling their transposition events.

## Physiological and pathological roles of distinct somatic mutation types in the brain

### SNVs and indels

Single-cell sequencing has revealed that mutation frequencies within individual cells were significantly higher than previously estimated, even exceeding germline mutation levels [195]. Notably, cancer studies have repeatedly identified SNVs and Indels in vulnerable hotspots of the genome [196]. These mutations are frequently enriched in transcriptionally active regions [40, 64], and they tend to occur in long genes—those over 100 kb in length—which often play key roles in synaptic formation and neuronal connectivity [197], as well as in genes associated with neurodegenerative disorders [198, 199]. In the human cortex, researchers have also detected a large number of Indels in genes associated with neurodegenerative diseases [198].

SNVs often inactivate genes, leading to reduced protein expression and impaired cellular function [200, 201]. Indels occurring in coding or regulatory regions frequently cause frameshift mutations, disrupting protein function and altering expression levels [202, 203]. Evidence suggests that this mutation accumulation contributes to transcriptional noise, which disrupts key signaling pathways and compromises cellular homeostasis [24, 204].

Researchers therefore propose that the accumulation of somatic mutations in neurons may underlie the development of neurodegenerative diseases [205]. Although approximately 30% of SNVs and Indels in the brain represent single-cell-specific mutations [198], and thus affect relatively few cells, pathogenic mutations may influence neighboring normal cells through inflammatory signaling, exacerbating disease progression [206]. Some have also hypothesized that these pathological effects could propagate through mechanisms similar to prion-like protein transfer [207].

Traditionally, scientists have linked age-related somatic mutations with aging and disease [48]. However, recent studies show that somatic mosaicism not only has detrimental effects but also promotes neuronal diversity by enabling different neuronal clones to perform specialized functions [208, 209]. SNVs may modulate early lineage expansion or restrict developmental potential, contributing to inter-individual variability in cortical composition and potentially regulating physiological processes during brain development [210, 211].

### SVs

SVs significantly alter gene expression patterns by reshaping chromatin architecture, disrupting cis-regulatory elements, or introducing novel regulatory sequences [212–215]. Similar to SNVs and Indels, studies in cancer have reported mutation hotspots for SVs [196]. In the brain, it has observed comparable patterns. DSBs, a key driver of SVs, frequently occur in neural stem and progenitor cells. It has identified 27 recurrently damaged gene clusters—including *Lsamp*—which may be particularly susceptible to replication stress, transcriptional activity, or oxidative damage [216–218].

During neurodevelopment, human stem cells exhibit substantial changes in *Alu* and *L1* recombination frequencies as they differentiate into neurons [70]. Low methylation levels during critical embryonic stages likely facilitate *L1* retrotransposition [219]. In adulthood, *L1* remains active in the hippocampus and plays a key role in memory encoding [220]. Inhibiting *L1* activity through lamivudine treatment or injection of *ORF1* and *ORF2* impairs long-term memory formation [220, 221]. Similarly, antisense oligonucleotides targeting *L1* reduce long-term memory in rats [222].

In non-pathological neurons, researchers have detected diverse *APP* cDNA variants, which may serve as genomic repositories for long-term synaptic information storage through integration into the genome [223]. However, this interpretation remains controversial, as some have suggested the findings may result from exogenous contamination rather than genuine genomic incorporation [224].

### mtDNA mutations

The mitochondrial genome encodes essential components of the electron transport chain and elements required for mitochondrial protein synthesis, including rRNAs and tRNAs [225, 226]. Its mutation rate far exceeds that of the nuclear genome [227]. mtDNA displays heteroplasmy, in which wild-type and mutant copies coexist; the proportion of mutant mtDNA generally correlates with clinical severity [227].

mtDNA mutations impair ATP production and damage organ and tissue function. Because the nervous system demands high energy, ATP depletion severely disrupts neuronal function and contributes to neurodegenerative disorders—a phenomenon observed in multiple mitochondrial diseases [228, 229]. In addition to directly affecting mitochondrial function, mtDNA mutations influence nuclear genome stability. Pathogenic mtDNA mutations can reshape global DNA methylation landscapes, further destabilizing cellular homeostasis [230].

Given the ubiquity of mtDNA mutations in the general population, researchers have begun to explore their potential physiological functions. Some studies suggest that certain mtDNA mutations may confer short-term protection against acute diseases [231]. Others hypothesize that somatic mtDNA mutations regulate neuroendocrine, metabolic, and inflammatory stress responses by modulating mitochondrial energy output and redox homeostasis [232].

## Somatic mutations in AD

### SNVs and indels in the AD brain

Early studies failed to detect an increased burden of somatic SNVs and Indels in neurons from AD patients, although they did report a positive correlation with age [233, 234]. More recent single-cell sequencing studies have revealed a significant increase in somatic SNVs and Indels in AD brains, particularly associated with mutational signatures Signature C and ID-A [34, 40, 54, 56]. In some neurons, the number of Indels can exceed several thousand [56].

**Fig. 3:**
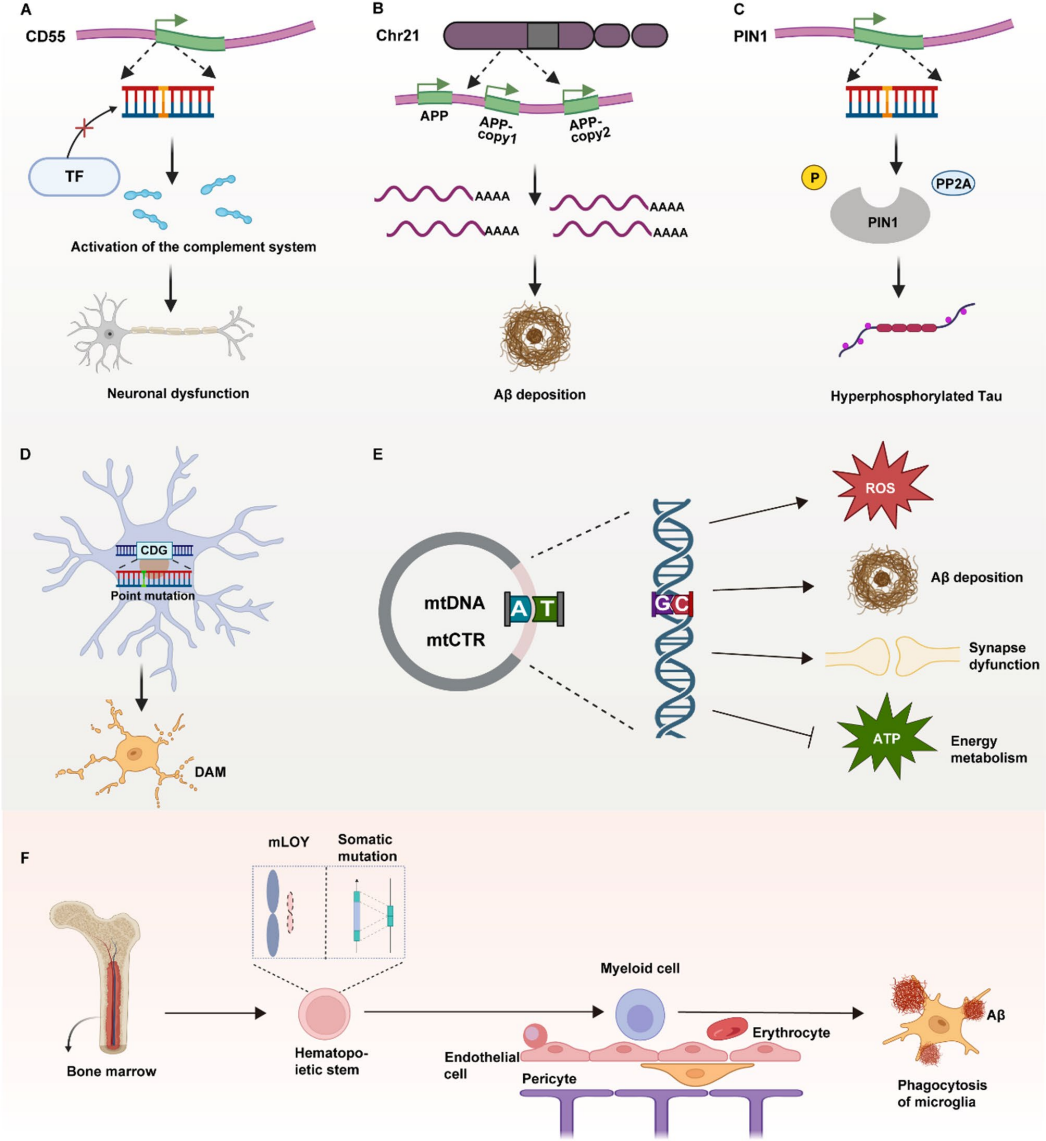
Impact of somatic mutations on AD pathology

In 2004, researchers identified a somatic *PSEN1* mutation (P436Q) in the cortical neurons—but not in peripheral blood—of a patient with SAD, providing early evidence that somatic mutations may contribute to AD pathogenesis [235]. However, Min et al. failed to detect any SNVs in the coding regions of *APP*, *PSEN1*, *PSEN2*, or *APOE* in 16 AD brain samples [236], suggesting that other forms of somatic mutations might play a broader role in disease progression. For example, studies show that somatic SNVs significantly enrich genes involved in the ubiquitin-dependent protein catabolic process in the AD cortex [34], a pathway closely associated with AD development [237]. In contrast, this enrichment is absent in regions like the cerebellum, which are less affected in AD [34]. Park et al. reported that pathogenic somatic SNVs in the AD hippocampus significantly enrich genes involved in the *PI3K-AKT*, *MAPK*, and *AMPK* pathways, which are related to tau hyperphosphorylation [234]. They identified a loss-of-function SNV in *PIN1*, an isomerase involved in these pathways [238, 239], and in vitro experiments confirmed that *PIN1* loss leads to tau hyperphosphorylation (Fig. 3C)[234].

In another study, researchers found that pathogenic somatic mutations in microglia from AD brains hyperactivate the *MAPK* pathway, initiating inflammatory cascades [240]. Microglia are developmentally categorized into yolk sac-derived microglia (YSMg) and monocyte-derived microglia (MoMg) [241], with YSMg being the predominant population under physiological conditions [242]. Somatic expression of *BRAF*^*V600E*^ in yolk sac erythromyeloid progenitors induces clonal expansion of mutant microglia and drives severe neurodegenerative pathology [27]. In AD, MoMg undergo significant expansion [243]. These cells originate from hematopoietic stem cells (HSCs), making them key targets for clonal hematopoiesis (CH), an age-related disorder characterized by the expansion of HSCs carrying cancer-associated somatic SNVs and Indels [244–246]. MoMg harboring CH mutations can promote neurodegeneration [241]. However, shared CH-associated mutations in peripheral and microglial cells do not necessarily indicate a monocytic origin, as these mutations may arise in multipotent progenitors or even earlier developmental stages [247].

Interestingly, AD brains also exhibit abundant microglia harboring CH-related SNVs and Indels, which shift their transcriptomic profiles toward disease-associated microglia (DAM) (Fig. 3D) [34]. Some studies even propose that CH-mutant HSC-derived MoMg may enhance Aβ phagocytosis, thereby exerting neuroprotective effects and slowing AD progression (Fig. 3F)[248]. This discrepancy may reflect: (i) fundamental differences between peripheral and CNS-acquired CH mutations, or (ii) AD-related blood-brain barrier disruption that allows excessive infiltration of mutant myeloid cells and triggers pathological inflammation.

Beyond mutations in protein-coding regions, studies have implicated non-coding SNVs and Indels in various diseases [36]. For example, specific miRNA mutations found in cancers can influence precursor structure, alter protein binding, and disrupt mRNA target recognition [249, 250]. Helgadottir et al. identified an SNV in the upstream regulatory region of *CD55* in the brain of an SAD patient. This mutation likely impairs transcription factor binding and disrupts gene expression (Fig. 3A) [251]. Under chronic inflammation, neurons express *CD55* to protect against excessive complement activation commonly observed in AD, thereby limiting inflammation-induced damage [252–254].

Somatic SNVs and Indels may not only drive AD development but also result from AD pathology itself. For example, individuals carrying the *APOE4* allele exhibit higher levels of sSNVs in brain tissues than non-carriers [34], suggesting a possible vicious cycle in which pathogenic somatic mutations promote AD progression, while genetic risk factors like *APOE4* further elevate mutation burdens in the brain.

### SVs in the AD brain

As previously discussed, SVs encompass various types of genomic alterations. Dileep et al. reported a higher burden of SVs, including gene fusions, in specific neurons from AD brains [111]. Aging fruit flies exhibit increased transposon activity [255, 256], but the evidence in AD remains mixed: while some studies found no significant increase in *L1* activity [257], others reported upregulated expression of transposable elements, including *L1* [186, 188]. Upregulated *L1* activity may increase transposition frequency and result in SVs.

In addition to SNVs and Indels, CH-related mutations also include mosaic loss of the mLOY [258]. Studies have demonstrated that increased mLOY burden in peripheral blood contributes to AD pathogenesis [259]. In the brain, mLOY primarily affects microglia and is significantly elevated in AD and AD-vulnerable brain regions [77]. These microglia exhibit enhanced pro-inflammatory phenotypes, altered phagocytosis and migration, and disrupted lipid metabolism [77].

While global aneuploidy levels do not differ significantly between AD and control brains, chromosome 21-specific aneuploidy increases tenfold in the AD cortex [260]. This chromosome harbors the *APP* gene, and increased *APP* copy number has been observed in frontal cortical neurons from SAD patients (Fig. 3B)[261]. Numerous studies have established a strong link between *APP* dosage and AD onset [262, 263]. Researchers have also identified fusion genes such as *Inka2-Atp5f1* and *Cdip1-Ubald1* in AD neurons [111]. Both *Inka2* and *Cdip1* are p53 targets, and their disruption by structural variations may induce transcriptional changes associated with cellular senescence [264–266], a process potentially relevant to AD-related neuronal aging.

### Somatic mutations in MtDNA in AD

Mitochondria play a crucial role in the aging process and the pathogenesis of AD. As early as the last century, scientists speculated that mutations in mitochondrial DNA contribute to aging and neurodegenerative diseases [267]. Recent studies have supported this notion and found that increased mutations in the mitochondrial DNA control region (mtCTR) reduce mitochondrial copy numbers and transcription in both AD and tumors [268, 269]. Interestingly, reduced mtDNA abundance impairs mitochondrial ATP synthesis [270] and contributes to AD pathology [271, 272]. Post-mortem analyses reveal that lower mtDNA copy numbers correlate with worse cognitive performance [273]. These findings suggest that increased mtCTR mutation load in AD reduces mtDNA abundance and impairs cognitive function. The catalytic subunit that Polg encodes is essential for mtDNA repair, and studies have shown that *PolG*^*D257A/D257A*^ mice, which express an error-prone DNA polymerase and generate numerous mtDNA mutations with age [274], produce a significant amount of 8-oxoG [275]. As previously mentioned, this form of guanine can lead to mismatching, highly correlated with Signature C in SNVs. Furthermore, work using this mutant mouse model has revealed that an increased mtDNA mutation load may exacerbate Aβ deposition and brain atrophy in APP/Ld mice [276]. Additional research indicates that mice carrying pathogenic deletion mutations in mtDNA exhibit impairments in long-term memory retention and impair spatial remote memory by downregulating α-CaMKII [277]. In summary, increased mtDNA mutations and reduced copy number drive mitochondrial dysfunction, which plays a pivotal role in aging and AD progression, with these alterations contributing to cognitive impairments and pathological features such as Aβ deposition and brain atrophy (Fig. 3E).

Somatic mutations accelerate the progression of AD through various mechanisms. A: Mutations in the *CD55* regulatory region may affect its expression, leading to a diminished neuroprotective effect and resulting in neuronal damage from complement activation. B: Copy number variations in the *APP* gene lead to increased production of Aβ, promoting its deposition. C: Mutations in the coding region of *PIN1* impair its function, facilitating Tau hyperphosphorylation. D: In microglia, mutations in CDG enhance the pro-inflammatory phenotype, contributing to neuronal injury. E: mtDNA mutations increase ROS production, Aβ deposition, disrupt energy metabolism, and damage synaptic function. Some somatic mutations, however, may offer protective effects; for example, F: Myeloid cells carrying mutations in genes implicated in clonal hematopoiesis (these mutations originate in hematopoietic stem/progenitor cells in the bone marrow) can infiltrate the brain, differentiate into microglia, and exert phagocytic activity, thereby protecting neurons.

## Prospective and future directions

Recent studies have identified somatic mutations in various genes as causative factors in certain neurodegenerative diseases. However, the pathogenesis of AD is complex, involving multiple cell types and genes. These mutations affect widespread brain regions and exhibit considerable cellular heterogeneity across different areas. These factors pose significant challenges in uncovering the mechanisms of AD.

Moreover, the accumulation of somatic mutations may actively contribute to the onset of sporadic AD. Certain factors in AD, such as Aβ, tau, and ROS, can increase the burden of somatic mutations through various mechanisms. Somatic mutations are more likely to occur in genes associated with AD, suggesting that these mutations may, in turn, accelerate the progression of the disease. We propose that somatic mutations and AD reinforce each other, forming a vicious cycle that accelerates disease progression.

Advances in technologies such as MDA, MALBAC [278], PTA [279], LIANTI [280], have enabled accurate amplification and sequencing of genetic material from individual cells. This technology is revolutionary for analyzing neurons with different mutation profiles, as it avoids missing somatic mutations due to low VAF [281]. However, single-cell sequencing still faces major challenges, including amplification artifacts, uneven coverage, and allelic dropout. To overcome these issues, various methods for calling single-cell mutations have been developed [282]. Researchers have also developed a range of tools to detect low-VAF somatic mutations in bulk sequencing data. Currently, third-generation sequencing technologies, such as single-molecule fluorescence sequencing [283] and nanopore sequencing [284], are rapidly advancing. Their long-read capabilities offer a more complete view of complex somatic mutations and structural variations. Furthermore, the emergence of multiple methodologies has enabled better detection of previously challenging somatic mutation types. For instance, INSurVeyor employs multi-algorithmic strategies combining reference-guided and de novo assembly approaches, achieving more sensitive detection of insertions [285]. Similarly, SVs detection tools integrating multiple methods have demonstrated superior performance compared to single-method detectors [286, 287].

Understanding the relationship between somatic mutations and AD holds great potential for elucidating disease mechanisms. First, identifying somatic mutations can help construct a more comprehensive network of AD pathological mechanisms. Second, by analyzing the unique or elevated somatic mutation characteristics in AD, we can map out the corresponding etiological factors of the disease. Finally, a detailed somatic mutation profile of AD can assist in identifying potential therapeutic targets. For certain important somatic mutations, gene editing technologies can be employed to restore their function. Additionally, These profiles can guide targeted therapies, including MAPK inhibitors and CBL-E3 ligase modulators [240], by pinpointing affected molecular pathways. Various technologies for editing mtDNA mutation sites have already emerged, significantly improving the associated clinical manifestations [288–290]. However, for nDNA, due to its complex structure and vast sequence, accurately editing specific loci remains a significant challenge.

## Data Availability

No datasets were generated or analysed during the current study.

## Abbreviations

AD: Alzheimer’s disease
PSEN1: Presenilin 1
APP: Amyloid precursor protein
mtDNA: Mitochondrial DNA
Aβ: Amyloid-beta
FAD: Familial AD
SAD: Sporadic AD
SNVs: Single nucleotide variants
SBS: Single base substitution
Indels: Small insertions and deletions
SVZ: Subventricular zone
SGZ: Subgranular zone
VAF: Variant allele frequency
NMF: Non-negative matrix factorization
8-oxo-G: 8-oxoguanine
OLs: Oligodendrocytes
PFC: Prefrontal cortex
DG: Dentate gyrus
MDA: Multiple displacement amplification
PTA: Primary Template-directed Amplification
MALBAC: Multiple annealing and looping-based amplification cycles
SVs: Structural variations
CNVs: Copy number variations
DSB: Double-strand break
NHEJ: Non-homologous DNA end joining
MMEJ: Microhomology-mediated end joining
TEs: Transposable elements
LTR: Long terminal repeat
NAHR: Non-allelic homologous recombination
ROS: Reactive oxygen species
nDNA: Nuclear DNA
DNA polγ: DNA polymeraseγ
mLOY: Mosaic loss of chromosome Y
MCI: Mild cognitive impairment
XP: Xeroderma pigmentosum
CS: Cockayne syndrome
AT: Ataxia-telangiectasia
BER: Base excision repair
NER: Nucleotide excision repair
ATM: Ataxia-telangiectasia mutated
UTR: Untranslated region
YY1: Yin Yang 1
ORF1: Open reading frame 1
CDGs: Cancer driver genes
DAM: Disease-associated microglia
mtCTR: Mitochondrial DNA control region
PCNA: Proliferating cell nuclear antigen
TOP1: Topoisomerase I
rNMP: Ribonucleotide misincorporation
rNTP: Ribonucleoside triphosphates
dNTP: Deoxyribonucleoside triphosphates
RER: Ribonucleotide excision repair
YSMg: Yolk sac-derived microglia
MoMg: Monocyte-derived microglia
CH: Clonal hematopoiesis
